# Egocentric Gesture Recognition Using 3D Convolutional Neural Networks for the Spatiotemporal Adaptation of Collaborative Robots

**DOI:** 10.3389/fnbot.2021.703545

**Published:** 2021-11-23

**Authors:** Dimitris Papanagiotou, Gavriela Senteri, Sotiris Manitsaris

**Affiliations:** Centre for Robotics, MINES ParisTech, PSL Université, Paris, France

**Keywords:** human-robot collaboration, gestures, actions, recognition, CNN, egocentric vision, collaborative robot, pose estimation

## Abstract

Collaborative robots are currently deployed in professional environments, in collaboration with professional human operators, helping to strike the right balance between mechanization and manual intervention in manufacturing processes required by Industry 4.0. In this paper, the contribution of gesture recognition and pose estimation to the smooth introduction of cobots into an industrial assembly line is described, with a view to performing actions in parallel with the human operators and enabling interaction between them. The proposed active vision system uses two RGB-D cameras that record different points of view of gestures and poses of the operator, to build an external perception layer for the robot that facilitates spatiotemporal adaptation, in accordance with the human's behavior. The use-case of this work is concerned with LCD TV assembly of an appliance manufacturer, comprising of two parts. The first part of the above-mentioned operation is assigned to a robot, strengthening the assembly line. The second part is assigned to a human operator. Gesture recognition, pose estimation, physical interaction, and sonic notification, create a multimodal human-robot interaction system. Five experiments are performed, to test if gesture recognition and pose estimation can reduce the cycle time and range of motion of the operator, respectively. Physical interaction is achieved using the force sensor of the cobot. Pose estimation through a skeleton-tracking algorithm provides the cobot with human pose information and makes it spatially adjustable. Sonic notification is added for the case of unexpected incidents. A real-time gesture recognition module is implemented through a Deep Learning architecture consisting of Convolutional layers, trained in an egocentric view and reducing the cycle time of the routine by almost 20%. This constitutes an added value in this work, as it affords the potential of recognizing gestures independently of the anthropometric characteristics and the background. Common metrics derived from the literature are used for the evaluation of the proposed system. The percentage of spatial adaptation of the cobot is proposed as a new KPI for a collaborative system and the opinion of the human operator is measured through a questionnaire that concerns the various affective states of the operator during the collaboration.

## 1. Introduction

Robots were first introduced to industrial environments in the mid-1950s and consequent advancements in the areas of perception of humans and of the environment, during the last few decades, have led to the evolution of a new area of research, named Human-Robot Interaction (HRI). The International Federation of Robotics (IFR)^1^ reports a record of 2.7 million industrial robots operating in factories around the world, which indicates an overall increase of 12% for the year 2020 alone.

Until quite recently, conventional automation of Industry 3.0 has been trying to insert more and more robots into production processes to perform repetitive and hazardous tasks which have traditionally been performed by humans. The translation to Industry 4.0, using means such as cyber-physical systems (CPS), cloud computing and Industrial Internet of Things (IIoT)s, aims to insert human-robot collaboration (HRC) frameworks into the manufacturing process. There are different categories of HRI, depending on the workspace, the aims, the working times of the robot and the operator.

The current work aims at the development of a Human-centered Artificial Intelligence perception layer of a robot, which is inserted in an industrial HRC scenario. Active vision through gesture recognition and pose estimation enables the spatiotemporal adaptation of the robot to each user. We focus on the insertion of a smaller, lightweight robot which facilitates HRC, without the need for physically separated workspaces. Different types of interaction are implemented and ultimately the goal of this paper is to evaluate their impact on both human-robot collaboration and user experience. On the way to safer and more effective HRC scenarios, touchless interaction is implemented.

Egocentric computer vision for action/gesture recognition unleashes great potential for touchless HRI. The proposed human egocentric system constitutes an initial step in active vision. It is not affected by some critical issues for active vision as the camera is unique, on the top of the operator, and moves according to operator's head motion. There is no change or motion of the camera for better field of vision. In addition, occlusion or limited resolution are improbable as the operator's actions are executed in front of her/his body. These actions are communicated to the robot so as to dynamically adapt its behavior. Therefore, both the temporal and spatial profile of the motion of the robot depend on the rhythm and the pose of the human operator, respectively. From an industrial point of view, the production cycle time becomes adaptable.

This paper presents a gesture recognition module based on 3D Convolutional Neural Networks (3DCNNs), trained on an egocentric view, for a natural collaboration between the human and the robot. Deep Learning (DL) is a field of Machine Learning (ML) with impressive results in pattern detection, speech recognition and many more applications and can provide the necessary robustness that an HRI scenario requires. The two hypotheses that are tested, are the reduction of cycle time of the assembly routine through the insertion of gesture recognition, as well as the improvement of the handover position via the implementation of pose estimation.

This paper consists of nine sections in total. Following the Introduction in section 1, section 2 presents human-robot interaction that can be achieved either physically or by touchless means. In section 3, the routine which was implemented, together with the experiments which are used to evaluate the contribution of the proposed modules (gesture recognition, pose estimation, sound notification) are described. In sections 4 and 5, the modules and their respective implementation methodologies are presented. section 6 describes the way that the robot performs and presents a variety of metrics that are commonly used for the evaluation of an HRI system. In section 7, each type of collaboration is described and evaluated, while, in section 8, future work perspectives emerging from the various types of collaboration are examined, with areas for future research suggested in Conclusion, in section 9.

## 2. State of the Art

Since the initial establishment of robots in industry, the aim has been to assist humans in their heavy-duty tasks, and to keep everyone safe at the same time. The limitations of robots, in this early period, in conjunction with the ever-increasing levels of safety which have had to be observed in industry, have served to create a somewhat primitive workplace for industrial robots. Traditionally, they have been installed in assembly-lines and have been assigned to undertaking the tasks which are repetitive, heavy-duty and dangerous for human operators, as described by Hentout et al. (2019). Regardless of their efficiency and velocity, the assembly-lines that use this type of robot have been lacking in flexibility, especially when the presence of a human operator is required.

Humans, on the other hand, have the flexibility and the intelligence to consider different approaches to solve a problem and can choose the best option from among a range of possibilities. They can then command robots to perform assigned tasks, since robots can be more precise and more consistent in performing repetitive and dangerous work. This collaboration between industrial robots and humans demonstrates that robots have the capabilities to ensure maximum efficiency in manufacturing and assembly; however, the evolution of technology, together with the ongoing automation of traditional manufacturing and industrial practices, has shown that there are many tasks which are too complex to be fully executed by robots, or are too financially burdensome to be fully automated.

This is the reason why the research agenda in the past few years has focused on creating appropriate industrial working environments, where robots and human operators can interact effectively. Nowadays, mixed environments are being created and industries aim to explore and create the ideal working environment through combining the cognitive skills of the human operators (intelligence, flexibility, and ability to act when confronted with unexpected events) with the ergonomic advantages of the robots (high precision, repeatability, and strength) (Prati et al., 2021).

The creation of mixed industrial environments, where humans and robots co-exist and work for a common goal reinforces the necessity of the insertion of cobots in manufacturing process. IFR in accordance with ISO 8373 describes two different types of robots (industrial and service). Cobots could be considered to be service robots, since they are intended to work alongside humans; however, there are different definitions of cobots, depending on the applications they are used for. In the beginning, a cobot was defined as “an apparatus and method for direct physical interaction between a person and a general purpose manipulator controlled by a computer” (Bicchi et al., 2008); however, due to the development of the sensors that cobots use and because of the way they interact with humans, this definition has evolved.

Active vision is mentioned as the capability of a robot to actively perceive the environment and obtain useful information for various tasks (Chen et al., 2011). It is used in plenty of use-cases such as collaborative robotics (Queralta et al., 2020) and industrial applications (Muhammad et al., 2017). The workflow of a typical active vision or perception system, includes view planning, motion planning, sensor scanning and map updating (Zeng et al., 2020). After each stage information is collected and update the status of the robot and its task goal.

In recent research, a cobot is referred to as a robot that has been designed and built to collaborate with humans (Schmidtler et al., 2015), or as a robot intended to physically interact with humans in a shared workspace (Colgate and Peshkin, 2010). For this reason, the discussion has shifted to Human-Robot Interaction and the way this interaction is achieved in each application.

### 2.1. Categories of Human Robot Interaction

HRI research has attracted the attention of numerous research domains. For this reason, HRI can be classified into many categories depending on the criteria that are used. Kopp et al. (2021) and El Zaatari et al. (2019) distinguish HRI as functioning on different levels, according to the workspace (separated or common), the working time/steps (sequential or simultaneous) and the aims (different or common) of the robot and the human operator respectively. At the lowest level, human and robot work alongside each other without a shared workspace (Long et al., 2018). They have neither common tasks, nor actions, nor intentions. Traditional industrial robots are used extensively in such cases. At the second level, however, the human and the robot share all or part of a workspace, they do not work on a part or on a machine at the same time. Unhelkar et al. (2020) name this type of collaboration as sequential, which implies that the human operator adapts to the rhythm and the orientation of the robot, since its velocity and its trajectories are pre-defined.

In a few industries, in recent years, humans and robots have been working on the same part or machine at the same time, and both are in motion (Cherubini et al., 2016). This level of interaction is called human-robot co-operation and requires advanced sensors and technology, like force/torque sensors or computer vision. Despite the sharing of workspace and aim, the human operator must adapt to the pre-defined temporal and spatial profile of the robot. That makes this type of interaction less natural than interaction between humans and, because of this, different types of communication and collaboration are established within the framework of Industry 4.0. Finally, at the upper level, the robot responds in real-time to the worker's motion which is called responsive HRC. The combination of artificial intelligence and high-tech sensors make robots able to adapt their rhythm and motion to unpredictable incidents and the anthropometric characteristics of the operator. The purpose of this category is the transformation of the robot, from being more than just a useful machine, to being a real collaborator.

Responsive Human-Robot Collaboration can be classified into physical (pHRC, Ajoudani et al., 2018) and touchless (tHRC, Khatib et al., 2017). pHRC can be divided into two different categories, depending on the intended purpose of the touching. On the one hand, there are operations which were intended to be without contact, but where instinctively the operator touches the robot. On the other hand, there are operations where the operator presses or touches the robot on purpose and the robot reacts in a particular way, depending on the amount and the direction of the operator's force. In the first case, the robot should perceive the presence and the velocity of the human operator inside its workspace and react correspondingly, either by reducing its velocity or protectively stopping its motion in order to avoid a collision, as noted by Michalos et al. (2015). In the second case, Bo et al. (2016) note that the robot can either be used as a tool which extends the capabilities of the human operator (strength, precision etc.), or can be taught by demonstration in order to be able to repeat a certain task precisely.

Long before the outbreak of Covid-19, which has necessitated social distancing, industries were using technologies that minimize the need for physical interaction among industrial workers, enabling device operation at a safe distance^2^. Contactless technology is a branch of control technology, which has as its aim the establishing of communication between computers/machines and human operators, without the need for any contact whatsoever. It relies on the interpretation of human movement and behavior, using ML algorithms and sensors, namely RGB-D cameras, thermal cameras or Inertia Measurement Units (IMUs, Zhang et al., 2020). The sensors and algorithms provide the machines/cobots with commands or instructions derived from the detection of facial patterns (Melinte and Vladareanu, 2020), voice translation (Gustavsson et al., 2017) and gesture recognition (El Makrini et al., 2017).

Contactless technology allows users to control digital or industrial systems, using their anthropometric characteristics or motion. It has gained a lot of attention in the gaming and medical worlds, as well as in other fields, such as the automotive and cultural industries. Human action recognition is one of the tools used to achieve contactless communication between a computer/machine and a human operator, and can be defined as the conversion of a human/humanoid movement or signal to a form understandable to a machine. Action recognition enables the human operator to interrelate with a machine in an environment characterized by the absence of means for physical interaction.

### 2.2. Movement-Based Implicit and Explicit Interaction

With a view to more natural HRC, the adaptation of robots, in accordance with the temporal and spatial profile of the human operator, has evolved into a very meaningful research topic. Humans can be involved, beyond their traditional offline role, as they can now interact with a cobot either explicitly or implicitly (El Zaatari et al., 2019). Explicit interaction, on the one hand, is what is referred to as direct communication between the robot and the human. Implicit interaction, on the other, involves an action (or practical behavior), which represents a message in itself, rather than a message being conveyed through language, codified gestures (such as a thumbs-up or nod of the head) (Gildert et al., 2018) or other non-verbal, sensorimotor forms of communication to send coordination signals (Pezzulo et al., 2013; Vesper and Sevdalis, 2020).

Temporal adaptation can be achieved either explicitly or implicitly. There is research where explicit interaction for temporal adaptation is achieved through the use of a button^3^ or a smartwatch (Michalos et al., 2018), thanks to which the operator can inform the robot that he/she has executed a task. However, if judged according to the previously-given definitions of HRI, this case matches more with human-robot co-operation, as the insertion of a button makes the interaction less natural. In the research of Cherubini et al. (2016), force feedback and pointing gestures are introduced as a means of HRC, in order to adapt the temporal profile of the robot and create hybrid interaction. In the present case, a totally implicit interaction is presented from our previous research (Coupet et al., 2019), which uses gesture recognition as a means to inform the robot about the percentage rate of completion of the human gesture, in order for it to react correspondingly. Such implicit interaction scenarios are also implemented outside of industrial workspaces, as described by Gabler et al. (2017) and Vogt et al. (2017).

The spatial adaptation of a robot to an industrial environment is commonly presented as collision avoidance between the robot and the human operator who share the same workspace (Mohammed et al., 2017; Safeea et al., 2019). Apart from their applications in industry, such adaptations are reported in other research, such as that of Canal et al. (2018), where the creation of a daily living assistant is presented. This research describes a cobot that is able to readjust its trajectories according to user movements and can thus handle incidents which are unpredictable. In the context of the present article, a spatiotemporal adaptation of a cobot, working according to the desired handover positions and rhythm of a human operator, is described. The goal of this research is to improve the perception of robots, using professional gesture recognition in cooperation with ergonomic parameters, with a view to creating a better and more natural HRC.

### 2.3. Machine Learning for Professional Gesture Recognition

A significant amount of scientific work aims at making machines smarter, improving their perception, enabling them to interpret human behavior, and to learn and react in a way similar to the human brain. In order to achieve these goals, solid results in the field of activity and, more specifically, in the field of gesture recognition are necessary, since this will permit more natural Human-Robot Collaboration (HRC). Indeed, an essential goal of the research community is the development of algorithms that can accurately recognize and understand human actions. Research on human action recognition focuses mainly on two strategies; namely, Pose- (Skeleton-) based recognition and Appearance-based recognition methods.

#### 2.3.1. Pose-Based Methods for Gesture Recognition

The main goal of pose-based methods is gesture recognition through the extraction of feature vectors which provide input to the corresponding ML algorithm. Essentially, those features are a set of coordinates able to describe the pose of a person and give explicit details about their position within a space. Pose estimation is usually performed using RGB-D cameras, such as the Kinect camera^4^, or optical hand tracking sensors such as the LeapMotion sensor^5^, algorithms, and modules such as Openpose (Cao et al., 2021), Alphapose (Fang et al., 2017), and Densepose (Güler et al., 2018), that use Deep Learning architectures themselves, performing either 2D or 3D pose estimation for both offline and online purposes, for the extraction of body joints. In general, recovering 3D pose from RGB images is considered more difficult than 2D pose estimation, due to the larger 3D pose space and other ambiguities. A number of factors can cause these ambiguities, such as body occlusions (Cheng et al., 2019), skin color, clothing, an overloaded background or quality of lighting (Rahmat et al., 2019).

Stochastic methods, such as Hidden Markov models (HMMs) (Borghi et al., 2016; Bui et al., 2018) and Random regression forests (Canavan et al., 2017), as well as DL methods, such as Recurrent Neural Networks (RNNs) (Shahroudy et al., 2016; Chalasani et al., 2018) have been used in various implementations for gesture classification. In the works cited, the aforementioned ML methods were used for the temporal correlation of the body features, leading to satisfactory classification results. Yan et al. (2018), in an attempt to create an algorithm that automatically learns both the spatial and temporal patterns from data, leading to a stronger generalization capability of the algorithm, propose a novel model of dynamic skeletons called Spatial-Temporal Graph Convolution Networks. Even though satisfactory results can be achieved, extracting features from data can lead to the loss of important information. The estimation of the human joints, and thus the skeletization of the whole body, must not only be absolutely accurate, but must be able to anticipate estimation problems caused by any of the factors mentioned previously (i.e., lighting, occlusions etc.). Thus, what constitute the challenges in these methods is not only the way that classification is performed, but also the way in which accurate pose-estimation is to be accomplished.

#### 2.3.2. Appearance-Based Methods for Gesture Recognition

In contrast with Pose-based recognition methods, Appearance-based ones consider visual cues (i.e., color and edges), to reach a gesture recognition result. Action recognition with these kinds of methods, can achieve results end-to-end, through mostly using sensors that extract visual information, such as RGB-D or thermal cameras. The end-to-end results are obtained by the hierarchical analysis of the characteristics of the visual input (edges, lines etc.) and algorithms, such as 3D CNNs (Tran et al., 2015), two stream fusion networks (Feichtenhofer et al., 2016) and inflated 3D convolution (I3D) (Carreira and Zisserman, 2017). One could say that the two-stream (RGB and optical flow) I3D models, based on 2D ConvNet Inflation, were a breakthrough in this field, as such models made it possible to learn seamless spatiotemporal feature extractors from videos, while leveraging successful ImageNet architecture designs and even their parameters.

There are many cases where the two categories of Pose-based recognition and Appearance-based recognition methods have been combined. Song et al. (2016) propose a multi-modal, multi-stream DL framework for egocentric activity recognition, using video and sensor data. They extend a multi-stream CNN to learn spatial and temporal features from egocentric videos, by using a multi-stream LSTM architecture to learn the features from multiple sensor streams (accelerometer, gyroscope etc.). Cao et al. (2017) perform egocentric gesture recognition, combining traditional CNN architectures with spatiotemporal transformer modules in order to address problems that arise from the global camera motion, caused by the spontaneous head movement of the device wearer. More specifically, a spatiotemporal transformer module (STTM) is proposed, that is able to transform 3D feature maps to a canonical view in both spatial and temporal dimensions. The challenge of capturing and recognizing images, from an egocentric view, lies in the fact that we can identify two parallel movements, that of the background and of the person themselves, and that of the camera that follows the motion of the head, with the motion of the head not always aligned to the motion of the rest of the body.

#### 2.3.3. Human-Robot Collaboration With Artificial Intelligence

Cobots are becoming ever more present in industrial environments, as an automated solution, enabling industrial workspaces to become more cost-effective, flexible, and ergonomic. For this to be accomplished successfully, cobots need to be equipped with tools that will make them adjust to the workspace and help the industrial operator, without creating an extra burden during the work process. These tools include ML algorithms, such as Markov chains or HMMs, and DL architectures, such as Convolutional Neural Networks (CNNs), Recurrent Neural Networks (RNNs) and deep Reinforcement Learning (RL) for gesture recognition, voice detection, working environment surveillance, to mention only a few.

Machine learning architectures such as Markov chains or HMMs are known for their applications in signal processing and pattern detection. They are used to estimate the probability of going from one state of a system to another and, therefore, lead to data classification. The limitation of these methods is connected to the fact that inserting images as input to be classified according to their state probabilities, demands preprocessing. This preprocessing concerns the extraction of features for the creation of vectors that will constitute the required input.

Many research projects (Liu and Hao, 2019; Sharkawy et al., 2019; Sharkawy et al., 2020) have used such approaches to detect a collision based on robot sensor stream data, or perform continuous gesture recognition (Tao and Liu, 2013). In order to enable a smooth Human-Robot collaboration, where the robot is able to synchronize, adapt its speed and detect any unexpected incident, Coupeté (2016) implements gesture recognition of professional gestures in an automotive assembly-line using Discrete HMMs and inertia sensors to finetune the results. Dröder et al. (2018) use an ML-enhanced robot control strategy, combining also a nearest neighbor approach, for obstacle detection in an HRC scenario. All of the cases mentioned above, require either the use of specific sensors that provide with-time-series, or involve time-consuming pre-processing, as previously discussed.

On the other hand, DL architectures, such as CNNs and RNNs, are widely used nowadays in finance, robotics and medicine. Such methods require a large amount of data in order to be trained properly and, in most cases, require a great deal of computational power and time. However, in some DL methods, such as CNNs, preprocessing is not necessary, ensuring there is no loss of information.

El-Shamouty et al. (2020), in trying to minimize the risk of accidents in HRC scenarios, propose a deep RL framework that encodes all the task and safety requirements of the scenario into RL settings, and also takes into account components such as the behavior of the human operator. Liu and Hao (2019) work on a scenario of multimodal CNNs and use a Leap Motion sensor for hand motion detection, as well as voice and body posture recognition. Amin et al. (2020) aim to upgrade safety and security in an HRC scenario, by using a combination of human action recognition and tactile perception in order to distinguish between intentional and incidental interactions if physical contact between human operators and cobots takes place. A Panda robot, along with a 3D-CNN for human action recognition and a 1D-CNN for contact-type detection, was deployed.

Most of the methods presented above are focused on specific factors (safety, accident prevention, fast response from a cobot in an HRC laboratory-implemented scenario), without considering all the limitations, as well as the spatiotemporal variations that might occur in a real-life scenario. Different users of the same set-up have different anthropometric characteristics and different behaviors when asked to perform the same action. The aim in the present work, however, is also to examine the contribution of an egocentric gesture recognition module with a Deep Learning architecture in an HRC industrial scenario.

**Figure 1:**
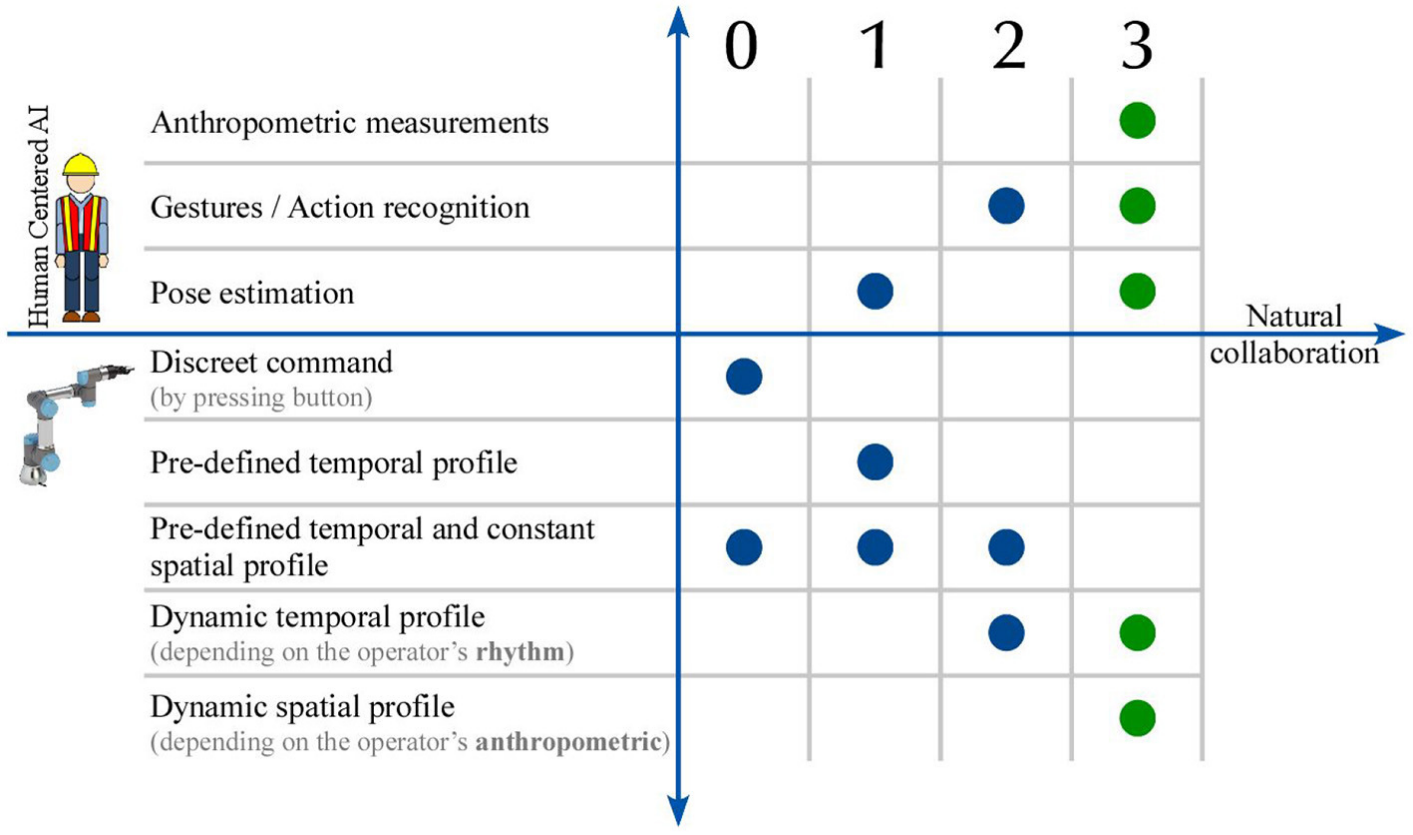
Evolution of Human-Robot Interaction (HRI) toward natural collaboration through human-centered Artificial Intelligence.

Figure 1 illustrates the potential of Human-centered AI in contributing toward a more natural HRC. The more anthropocentric the information that is extracted, the richer the perception of the robot is. The more its perception is enriched, the more it can predict human actions. In order for the robot to collaborate with the human, it has to understand not only its tasks but also human actions and intentions. At the beginning (level 0), the introduction of traditional industrial robots is the baseline, and the most common case in industry currently. There is no interaction between the robot and the operator and the robot completes a task very quickly and precisely. The first step toward interaction (level 1) was achieved by giving the robot information about the human's presence inside its workspace. Both spatial and temporal profiles remain constant and predefined, but when it perceives that an operator is inside its workspace, it reacts either by protectively stopping its motion or by reducing its velocity. Moreover, the human action and gesture recognition (level 2), converts the temporal profile to dynamic, adapting to the operator's rhythm. In the present research, the development of a dynamic spatiotemporal HRC framework is presented, receiving the human's actions and poses as input parameters (level 3).

## 3. Pilot Scenario

The use-case that was used for this research was derived from industry and, in particular, from Arçelik's TV assembly factory. In the actual assembly line, the task is executed manually by two different operators. The first operator has the task of picking up the electronic cards and placing them on the TV panel. The electronic cards are divided into two different types: the power supply (PSU) and the main board (chassis), that are located in two different boxes next to the conveyor belt. The second operator is responsible for the screwing of the cards onto the TV. The insertion of a temporal and spatial adjustable cobot, which can perform the first part of the operation, is proposed.

Factories in the Industry 4.0 era need the high efficiency and repeatability of the robots, together with the flexibility and variety of products that a human operator can provide. The parallel operation of a cobot and an operator on an assembly line was examined. The experiments were as follows:

1. Physical Interaction
2. Physical Interaction and Spatial adaptation (Operator's pose estimation)
3. Physical Interaction, Spatial adaptation (Operator's pose estimation), and Sound notification
4. Physical Interaction and Gesture recognition
5. Combination of spatiotemporal adaptation and sound feedback

Initially, the operator interacts with the robot only physically (pHRC). This is accomplished through a Force Sensor (FS) which is placed on the robotic arm, just above the end-effector (Gripper). Every time the operator finishes with a task, s/he presses the FS in order to inform the robot and make it advance to the next position. The operator presses the FS to start the routine. When the robot grasps the card, it brings the card to a particular position. Then, the operator presses the FS again to release the card and the robot advances to a waiting position. The operator decides if the card is functional or not and presses the FS accordingly. If the FS is pressed on the horizontal axis, the card is not functional and the robotic arm returns to take the next card from the same box. If otherwise, and the card is functional, the operator presses the FS at the vertical axis, as always, and the robotic arm continues and grasps a card from the second box. When the operator takes the first card, s/he places it on the TV board and s/he screws it in place. The same procedure is followed also for the second card and when it is well-positioned on the TV, the operator presses the FS to inform the robot that the routine is finished.

The physical interaction that has just been described is complemented with a pose estimation module, during the second experiment. The robotic arm does not place the cards in a particular position, as previously, but instead is spatially adapted to the anthropometric characteristics of each operator. This procedure improves the pose of the operator ergonomically. The skeleton of the operator is extracted, and the position and velocity of each wrist is recorded. When the human's hand is motionless and in a position which is reachable for the robot, the robotic arm records it and approaches it holding the card. A natural HRC also demands the exchange of information. Thus, for the third experiment, a sonic notification is inserted. This notification is activated when the operator asks for the card at a position that is not reachable for the robot.

Gesture recognition is implemented in the fourth experiment. Physical interaction is used only for the release of the card. Each card is delivered to a particular position, with the aim of evaluating the added value of the gesture recognition module in the HRC scenario. The camera that records the operator's gestures is placed on the operator's helmet, thus offering an egocentric view. The final experiment brings all of the 4 modules together. Physical interaction is used for the release of the card, pose estimation for the spatial adaptation of the robotic arm, with sound notification and gesture recognition used in the ways previously referred to. The aim of the final experiment is the evaluation of all the modalities together, in order to see what the positive contribution is for the human operator.

**Figure 2:**
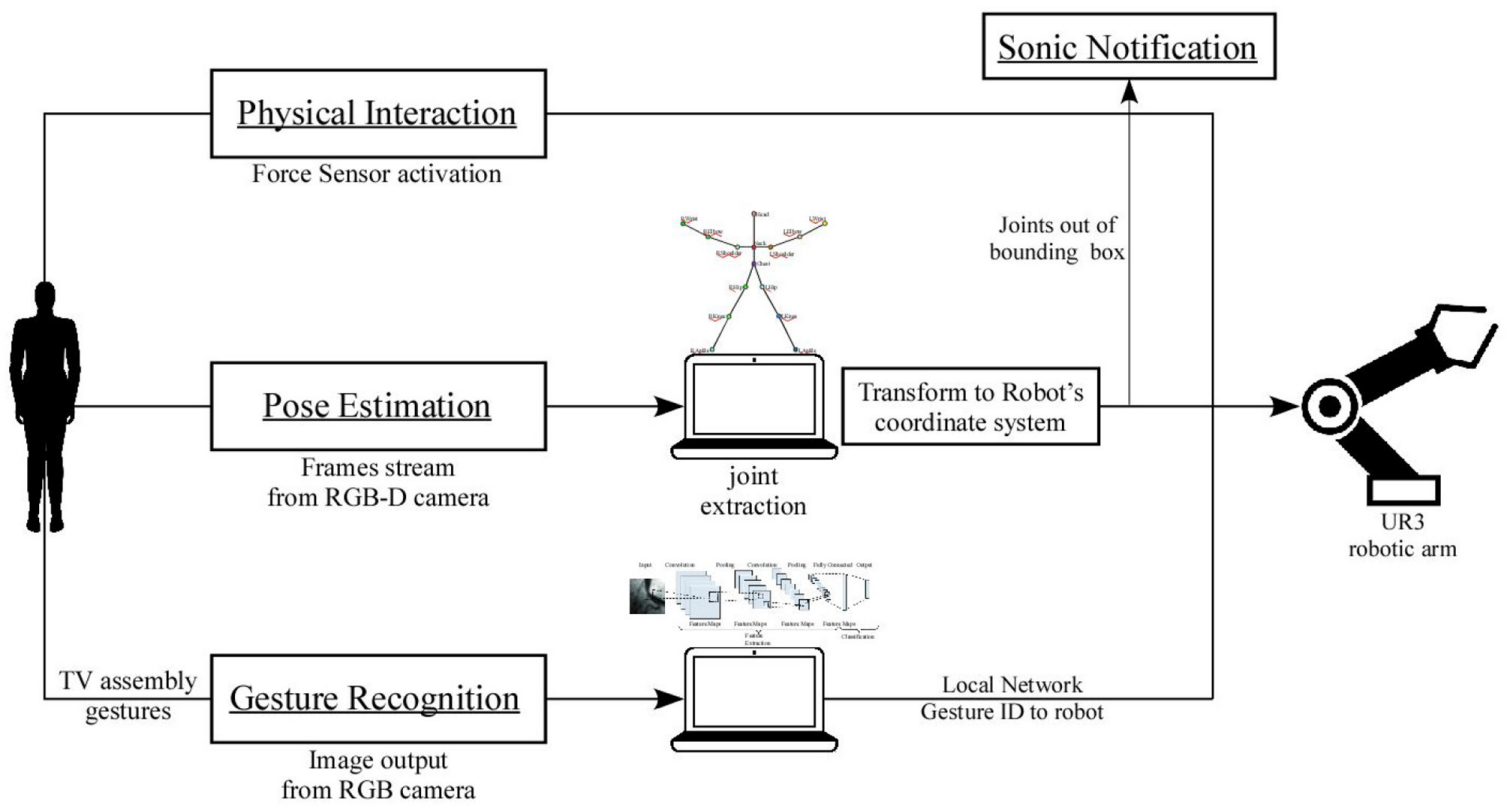
Architecture of the system.

Through the execution of the aforementioned five experiments, this research aims to evaluate the dynamic temporal profile that is achieved through the implementation of gesture recognition and the dynamic spatial profile that is achieved through the implementation of pose estimation. In addition, every experiment is executed twice, in order to indicate the compliance of the robot to unpredicted incidents (actions not corresponding with the work sequence). In Figure 2, the architecture of the system is presented. Physical interaction demands that the operator stop his/her task in order to inform the robotic arm about the work sequence. The cycle time is therefore expected to increase. The insertion of pose estimation is expected to improve the handover position of the card for each user; however, it will necessarily increase the cycle time because of the path calculation for the position of the operator's hand. Sonic notification is supposed to decrease the average cycle time, as each operator knows where to place the hand in order to ask for the card. Adding gesture recognition will reduce the cycle time as the operator interacts more implicitly with the robotic arm in comparison with other types of interaction. Thus, it is expected that experiments that contain gesture recognition will improve the naturalness of the HRC scenario, with users' responses gathered via questionnaires. The hypotheses that are extracted from the above expectations are the following:

**H1:** Can gesture recognition facilitate the temporal adaptation of the robot for reducing the cycle time in assembly lines?
**H2:** Can human pose estimation facilitate the spatial adaptation of the robot for reducing the range of motion of the operator and improving the handover position?

## 4. Pose Estimation of Handover Position for Robotic Arm

**Figure 3:**
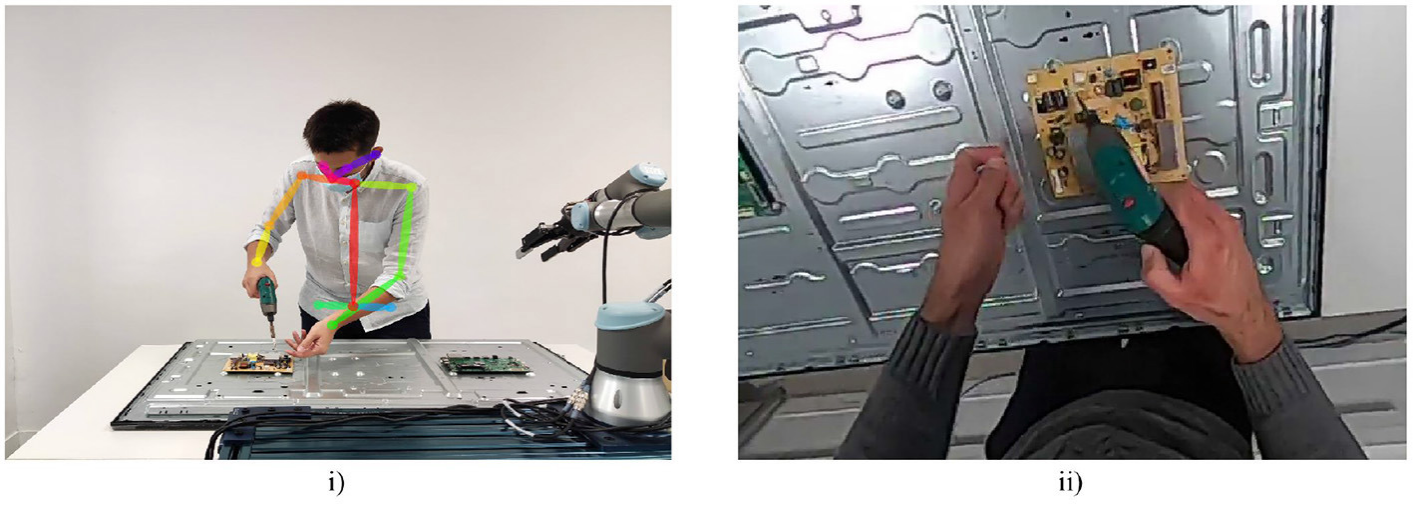
**(i)** View of the experimental setup from the camera that is used for pose estimation. **(ii)** Egocentric view of the experimental setup from the camera that is placed on the head of the user.

During the execution of the experiments “*Pose Estimation*”, “*Sonic Notification*”, and “*Combination*”, pose estimation is used as a mean of interaction between human and robot. The OpenPose^6^ framework is used for the skeleton extraction of the operator. This framework detects the body key points on RGB images and concludes with the extraction of 2D positions for each body joint, using DL architectures. Pose estimation, in the context of these experiments, was used both to estimate the position of the operator's right hand and to calculate its velocity. The coordinates of the right wrist, as extracted from the framework, are used. The camera that is used for the pose estimation is placed parallel to the operator, next to the conveyor belt. The framework extracts the position of the wrist in the image frame counted in pixels (X,Y) and an estimation of the distance on the Z axis is counted in meters. The procedure of providing the robot with the coordinates of the operator's wrist consists of two steps:

1. The first step is the conversion of the camera pixels to meters. Initially, the Intel-RealSense RGB-D camera is positioned so that the X and Y axes of the camera are parallel to the X and Z axes of the robot, accordingly. Using the parameters of the RGB-D camera that was used (focal length, principal point and distortion coefficients) it was possible to convert pixels to meters for each different depth value. The equations that were used are the following:
  (1) $$\begin{array}{l} x=\frac{\left(X-c_{x}\right)*z}{f_{x}}\;y=\frac{\left(Y-c_{y}\right)*z}{f_{y}} \end{array}$$
  Where c_x_, c_y_ is the central - principal point of the camera (956, 538) and f_x_, f_y_ is the focal point of the camera (973, 973). The camera that was used has no distortion coefficient.
2. As the position of the operator's wrist was defined in meters for the coordinate system (CS) of the camera (X_C_, Y_C_, Z_C_), the second step was the transformation of this CS to the robot CS. For this transformation, the homogeneous transformation matrix was used. For the X axis there is only transfer for d_1_, for the Y axis there is rotation of 90^o^ and transfer for d_2_, and for the Z axis there is rotation of 90^o^ and transfer for d_3_. Using the direction cosines of the initial point of camera CS to robot CS, the homogeneous transformation matrix is calculated and presented in the following equation:
  (2) $$\begin{array}{l} \left[\begin{array}{c} X_{R}\\ Y_{R}\\ Z_{R}\\ 0 \end{array}\right]=\left[\begin{array}{cccc} \cos\left(X_{C},X_{R}\right) & cos\left(Y_{C},X_{R}\right) & cos\left(Z_{C},X_{R}\right) & d_{1}\\ \cos\left(X_{C},Y_{R}\right) & cos\left(Y_{C},Y_{R}\right) & cos\left(Z_{C},Y_{R}\right) & d_{2}\\ \cos\left(X_{C},Z_{R}\right) & cos\left(Y_{C},Z_{R}\right) & cos\left(Z_{C},Z_{R}\right) & d_{3}\\ 0 & 0 & 0 & 1 \end{array}\right]\times \left[\begin{array}{c} X_{C}\\ Y_{C}\\ Z_{C}\\ 1 \end{array}\right]\\ =\left[\begin{array}{cccc} 1 & 0 & 0 & d_{1}\\ 0 & 0 & -1 & d_{2}\\ 0 & -1 & 0 & d_{3}\\ 0 & 0 & 0 & 1 \end{array}\right]\times \left[\begin{array}{c} X_{C}\\ Y_{C}\\ Z_{C}\\ 1 \end{array}\right] \end{array}$$
  Figure 3 shows the experimental setup during the execution of the experiments. Figure 3i presents the view of the camera that is used for the pose estimation. The skeleton that is executed through OpenPose during the 2nd, 3rd, and 5th experiment is demonstrated. In the meantime in Figure 3ii the egocentric view that is used for gesture recognition during the 4th and 5th experiment is presented.

## 5. Egocentric Gesture Recognition Using 3DCNNs

For the temporal adaptation of the cobot to the behavior of the human operator, a gesture recognition module was used in the experiments “*Gesture Recognition*” and “*Combination*”, which are described in detail below. Briefly, the gestures and postures of different human operators, during the TV assembly routine in an assembly line, were captured with a GoPro RGB camera, segmented and used for the training of a Deep Neural network with Convolutional Layers. The goal of this module was the exploration of the contribution of gesture recognition to an HRC professional scenario. The initial step for this module was the creation of a collaboration protocol between the human operator and the cobot. The parts of the use-case described that included decision-making, were assigned to the human operator, and those that did not, were assigned to the cobot. The gesture recognition results were sent as IDs to the cobot, which interpreted them and acted according to the defined protocol.

### 5.1. Network Architecture

The DL method used for egocentric gesture recognition in this work was 3D Convolutional Neural Networks (3DCNNs). 3DCNNs are the 3D equivalent of 2DCNNs, taking as input a 3D volume or a sequence of 2D frames. Image sequences with a size of c × l × h × w were used, where c was the number of channels, l was length in number of frames, h and w were the height and width of the frame, respectively. We also refer to 3D convolution and pooling kernel size by d × k × k, where d was kernel temporal depth and k was kernel spatial size. All image frames were resized to 84 × 48, so the input dimensions were finally 5 × 84 × 48 × 3. The network used had 6 convolution layers and 3 pooling layers, 1 fully-connected layer and a softmax loss layer to predict action labels. The number of filters for 4 convolution layers from 1 to 6 were 32, 32, 64, 64, 64, and 64, respectively. All convolution kernels had a size of 3 × 3 × 3, where *d* = 3, *k* = 3. All of these convolution layers were applied with appropriate padding (both spatial and temporal) and stride 1; thus, there was no change in terms of the size from the input to the output of these convolution layers. The standard ReLu activation function was used. All pooling layers were max pooling, with kernel size 3 × 3 × 3. The fully-connected layers had 512 outputs and dropout was not used. The output of the network was a softmax with 11 nodes, like the number of the gesture and pose classes. This network proved to be the most effective, in terms of recognition accuracy, after many experiments with network parameters and layers were performed.

### 5.2. Industrial Dataset and Gestural Vocabulary

The performance of 3DCNNs was evaluated by recording an egocentric dataset, inspired by an industrial TV assembly scenario. The main routine in assembling a TV is separated into sub-tasks, performed by either a human operator or a robotic arm. The objects that are involved in the assembly routine are a TV frame and two TV cards, one green and one gold. The human operator only performs gestures to interact with the robotic arm, while physical interaction (activation of the force torque sensor) is used only for the TV cards to be released by the gripper of the cobot.

**Figure 4:**
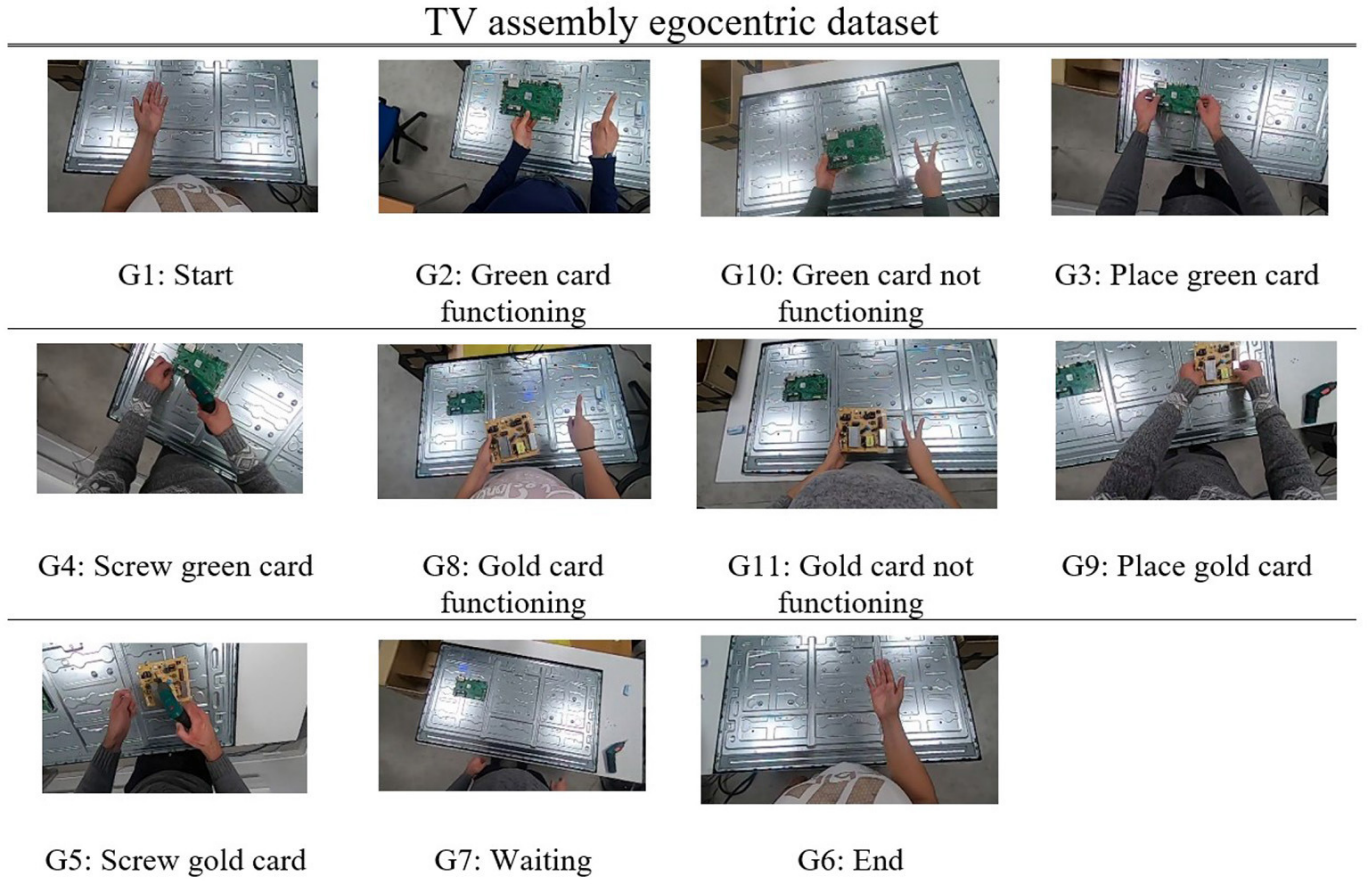
Presentation of the TV assembly dataset, consisting of 11 classes in total, 6 gestures, and 5 postures.

The dataset includes RGB sequences of images recorded at a resolution of 848 × 480 and 20 frames per second, presenting 13 users performing six different gestures that correspond to six different commands. These commands, given to the robotic agent by the human operator, along with five postures that were captured during the TV assembly routine, consist of a total of 11 classes, which are used as input to the classification algorithm. The gestural vocabulary is given in Figure 4.

Gestures are performed in a predefined working space, with a conveyor between the robotic agent and the human operator. A GoPro camera^7^ is mounted with a headband on the head of the operator, providing an egocentric view of the TV assembly process. There are two main challenges connected to capturing a dataset from an egocentric view. The first challenge concerns the “double” movement of the hands and the head. The hands of the operator move during the execution of the gesture, while the camera moves along with the head, and is therefore not always in accordance with the hands. The second challenge concerns the fact that due to the short distance, from the camera to the hands, and the field-of-view the camera has, the hands are usually prominent in the frame, but can also be partly, or even totally, out of the field-of-view.

More specifically, during the performance of the TV assembly scenario, the operator performs Gesture 1 (G1) to indicate the start of the assembly routine to the cobot. The cobot goes above the box with the TV cards, then toward the green card, takes it and hands it to the operator, who checks the card for functionality problems. In cases where this particular card is not functional (e.g., is broken, or has a missing part) the human operator performs Gesture 10 (G10) to notify the cobot, which in turn fetches the next green card. The operator verifies that the new green card functions and performs Gesture 2 (G2) to confirm the functionality of the card to the cobot. The operator places the green card (Posture G3) on the TV frame and starts screwing it in place (Posture G4). At the same time, the cobot approaches the gold card and gives it to the operator, as soon as the screwing procedure with the green card has finished. The operator then performs Gesture 8 or 11 (G8 or G11), depending on whether the gold card is functional or not. The above steps are repeated until the two cards are placed appropriately on the TV frame, and the TV is assembled. Finally, the human operator performs Gesture 6 (G6) to confirm the end of the assembly routine, until a new one starts again with Gesture 1 (G1). The captured gestures have the same duration, on average, apart from G4 and G5, during which the operator screws the green and the gold cards respectively.

**Figure 5:**
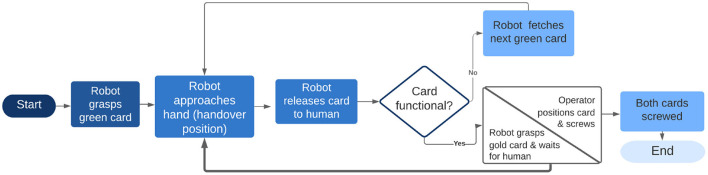
Work-flow of the HRC system.

To ensure the safety of the human operator, errors must be avoided; thus, two control layers were employed in decision-making. The recognized gesture ID was taken into consideration only if the same recognition accuracy result, with a probability of 100%, was extracted for twenty consecutive frames. The time between the capture of the frame, up to the correct classification of a gesture, was between 0 and 800 ms, thus leading to the conclusion that no important latency was observed during the performance of the HRC scenario. The extracted recognition result was transformed to an ID from 1 to 11 and the result was then sent to the cobot, through the use of a UDP communication protocol. At this point, the second layer of security was added. The thought behind this specific layer was based on the idea of a specific sequence performed during assembling a TV, without any important variations to be taken into consideration. Thus, the received accuracy result was checked by the cobot and was accepted only in cases where it corresponded with the expected gesture ID that was defined according to the work-flow and the scenario presented in Figure 5.

### 5.3. Gesture Recognition Results

For the evaluation of the performance of the gesture recognition algorithm and the proposed methodology, the metrics of *accuracy* and *f* − *score* were calculated. The *f* − *score* metric is derived by a combination of the metrics *recall* and *precision*. Those metrics are defined as shown below:


(3) $$\begin{array}{l} precision=\frac{\#\left(true\text{\_}positives\right)}{\#\left(true\text{\_}positives\right)+\#\left(false\text{\_}positives\right)} \end{array}$$

(4) $$\begin{array}{l} recall=\frac{\#\left(true\text{\_}positives\right)}{\#\left(true\text{\_}positives\right)+\#\left(false\text{\_}negatives\right)} \end{array}$$

(5) $$\begin{array}{l} f-score=2\frac{precision*recall}{precision+recall} \end{array}$$

Concerning the *accuracy*, if ŷ_*i*_ is the predicted value of the *i*-th sample and *y*_*i*_ is the corresponding true value, then the fraction of correct predictions over *n*_samples_ is defined as:


(6) $$\begin{array}{l} \text{accuracy}\left(y,ŷ\right)=\frac{1}{n_{\text{samples}}}\overset{n_{\text{samples}}-1}{\underset{i=0}{\sum }}1\left({ŷ}_{i}=y_{i}\right) \end{array}$$

The network presented was initially trained on the TV assembly custom dataset that was created as part of this work. The dataset was split into training and validation sets with a ratio of 80:20. The network was trained from scratch with a batch size of 32 frames and an Adam optimizer for 40 epochs. The accuracy results for offline gesture recognition, with a sliding window of 5 frames can be found in Table 1.

**Table 1:** Recognition accuracy and f-score with and without transfer learning.

		**Accuracy (%)**	**F-score (%)**
Test with no new users	No transfer learning	99.8	99.8
	Transfer learning	99.9	99.8
Test with new users	No transfer learning	84.68	60
	Transfer learning, 40 epochs	95.7	97.2
	Transfer learning, early stopping	98.5	98.6

**Figure 6:**
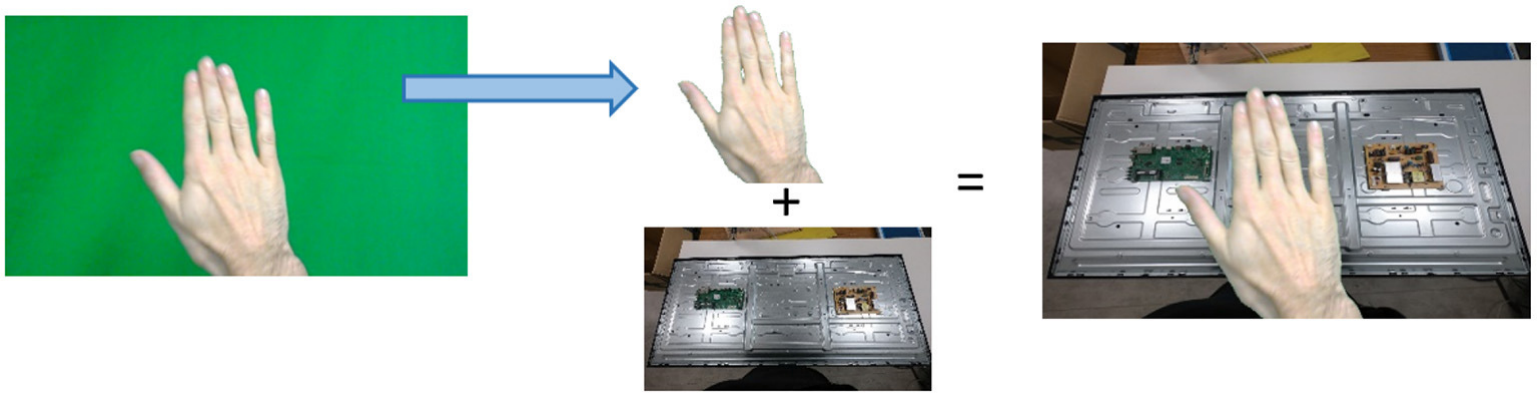
Preparation process of the dataset used for transfer learning.

After performing the same experiment, using only new users to whose gestures the recognition module was not trained (Table 1), it was observed that the network possibly needed to be trained to a larger amount of data in order to be able to distinguish the differences between the hands of the operator for each gesture. For this to be achieved, an egocentric gestural dataset, that was created during the work of Chalasani et al. (2018), was used for transfer learning. The dataset consisted of 10 classes of gestures captured in an egocentric view in front of a green background. The specific dataset included three iterations per user, for 22 users in total. Even though the size of this dataset cannot be considered appropriate for transfer learning, it had the advantage of being easily customized and turned into a larger dataset. In order for this to be achieved, around 100 images that provided a view of the TV assembly background (TV frame and TV cards), from different angles, were recorded. The green background of the original dataset was removed and replaced by a custom background, leading to a new, larger dataset, to be used for transfer learning. The process involved in the preparation of this dataset is shown in Figure 6.

**Figure 7:**
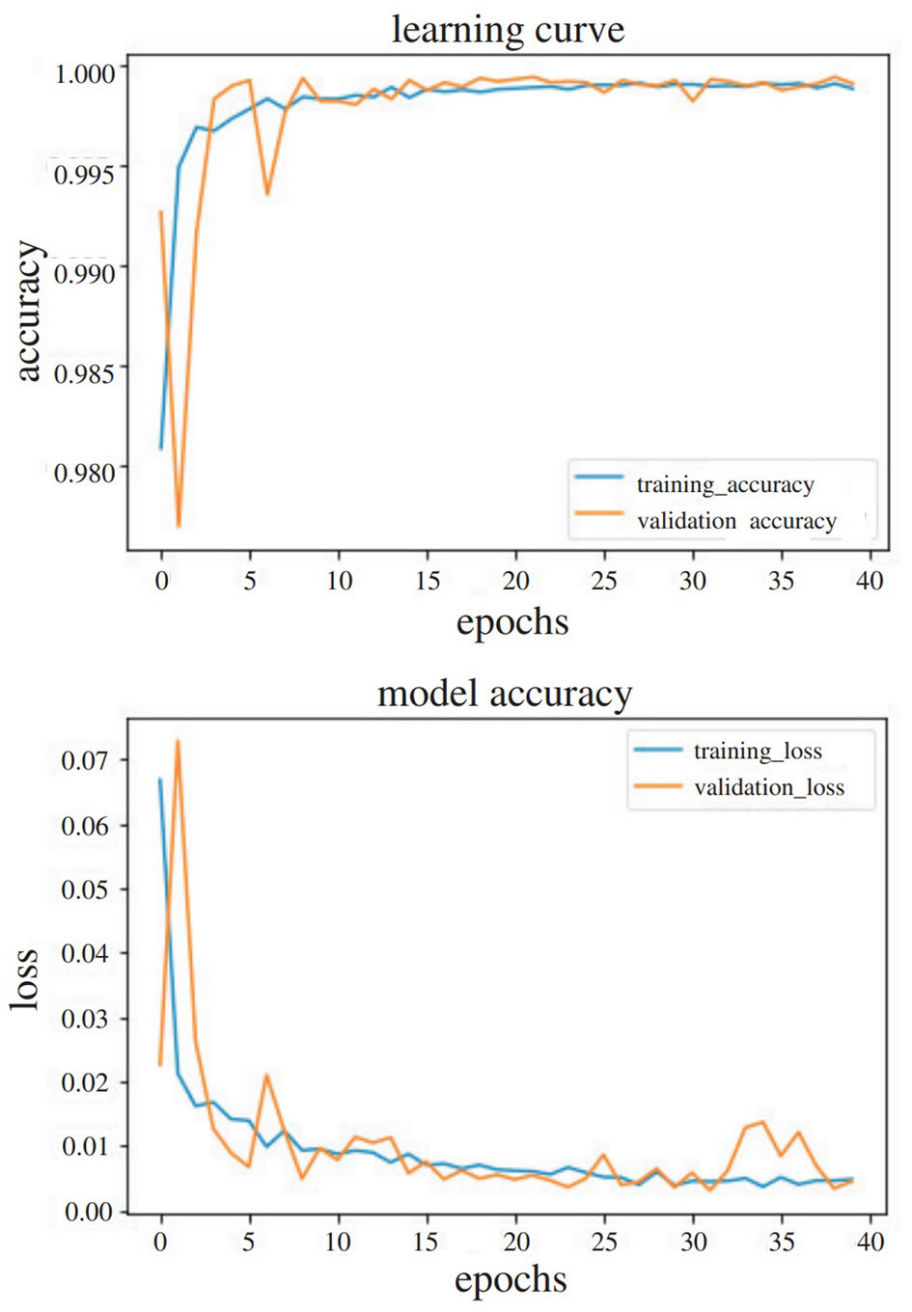
Accuracy and loss diagrams of the experiment with transfer learning, trained for 40 epochs in total.

To reach the final number of layers to be frozen, several experiments were performed. It was noticed that freezing network layers did not improve the recognition accuracy results, so after the initial training of the network with the improved dataset from Chalasani et al. (2018), the network was retrained, using the egocentric TV assembly dataset. The 80:20 approach was used again, and the stratification parameter was deployed to split it in such a way that the proportion of values, in the training set, would be the same as the proportion of values in the test set, leading to a balanced proportion in the classes within each. The recognition accuracy results, along with the f-score, with both the 80:20 approach and the testing of the network with completely new users, are shown in Table 1. Two diagrams of the accuracy and loss for an experiment using transfer learning to perform gesture recognition, with 40 epochs in total, with the 80:20 method is shown in Figure 7 for the visualization of the convergence of the training and testing phases.

**Figure 8:**
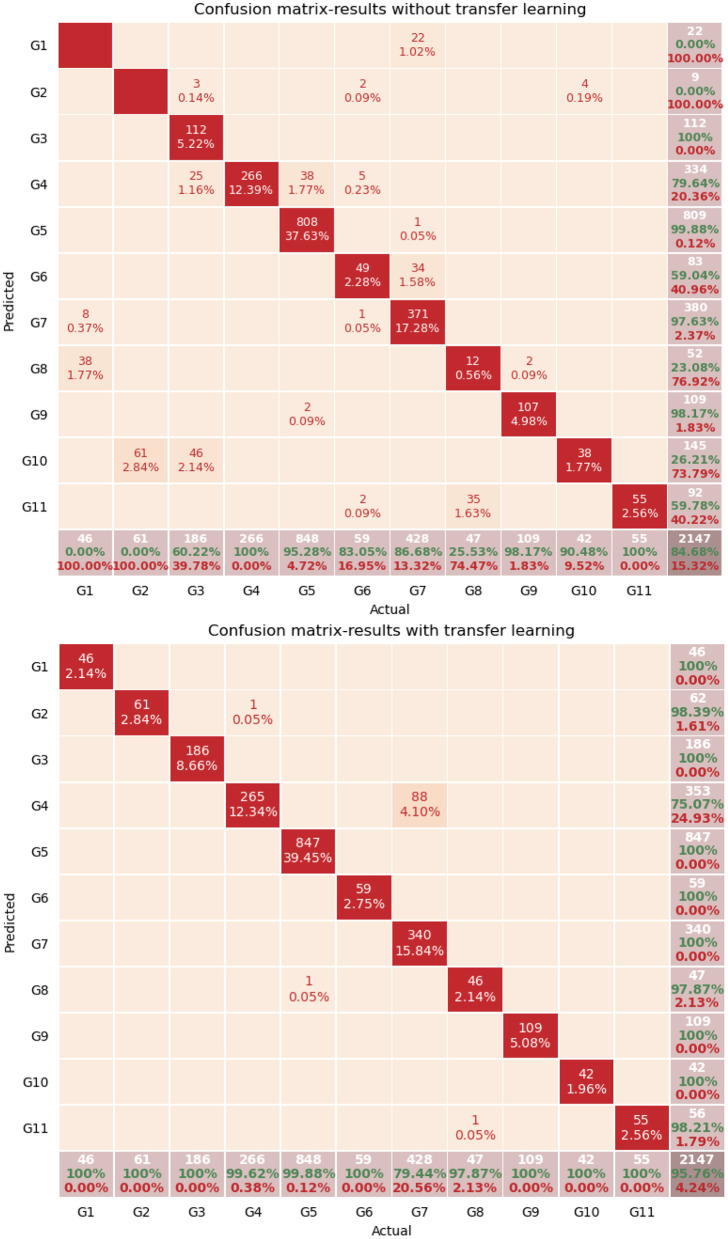
Confusion matrices with only new users in the test set. Without transfer learning **(top)** and with transfer learning **(bottom)**.

It was thus observed that transfer learning led to an improvement of 11% in the accuracy results, in cases where new users were introduced to the dataset, which is rather significant. After running the same experiment, using early stopping, the accuracy increased to 98.5%. Also, in Figure 8, the confusion matrices are presented with only new users in the testing set without the use of transfer learning (above) and with transfer learning (below).

In the two confusion matrices presented, a significant total improvement of 11% is observed, as already mentioned. More specifically, for each gesture, in the case where transfer learning was not used, G1 and G2 were not recognized correctly at all, while when transfer learning is used, the recognition level rises to 100%. Even if these gestures are considered as simple and rather static, transfer learning was required for the 3DCNN network to be able to perform accurate recognition. Concerning G5 (Screw gold card) and G9 (Place gold card), satisfactory results can be observed even without transfer learning, which can be explained by the fact that these two classes have the characteristic of the introduction of the gold card, which makes them much more discrete for the network than G1, G2, G3, G4, and G10.

**Figure 9:**
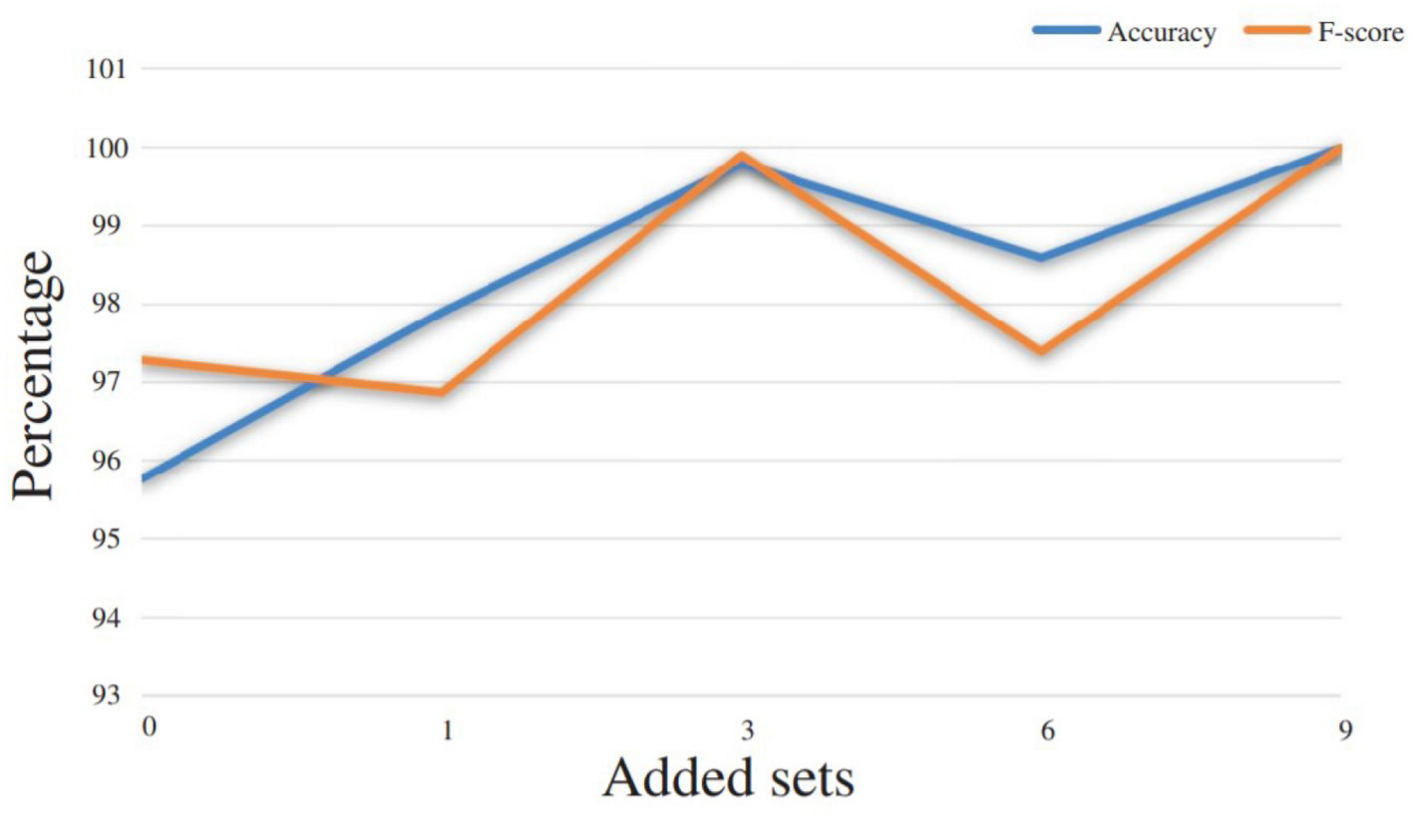
Performance improvement according to the number of gesture examples that are added in the training set and provided by a given user.

We can indeed foresee that when the learning base contains examples of an operator's gestures, his/her future gestures will be better recognized by the system. However, since the implementation of 3DCNNs is a method with high computational time demands, one of the goals of this work is to examine if the proposed gesture recognition module can be used in the assembly-plant directly, without any further training. At the same time, we had to ask ourselves how many iterations of the same operator were necessary in order to have an improvement in the recognition rate. In Table 2 and Figure 9, the improvement rates in recognition accuracy are presented in the cases where 1, 3, 6, or 9 iterations of the test user were added in the training phase. The baseline for these experiments is the result extracted when there are no iterations for this operator in the training set. At that point, a recognition accuracy of 95.76% was achieved, leading to the conclusion that, indeed, the proposed gesture recognition module could be used in an assembly line, without the need for it to be trained with samples from each new human operator. The rest of the results extracted provide an idea of what can be deemed a sufficient amount of data to be used in the training phase for the desired recognition results. In this particular case, it was the number of 3 sets that gave the best results and reached an accuracy level of up to 99.8%.

**Table 2:** Contribution of the number of sets from the new user in improving recognition accuracy.

	**Number of added sets**			
Improvement rates of recognition	1	3	6	9
	+2.2%	+1.9%	+0%	+0.2%

Other experiments performed using the same network architecture, but with a TV assembly dataset recorded not from an egocentric, but from a top view, provided results that reached up to 96% with an 80–20 approach. Thus, the results with only new users in the test set were much lower than the ones provided in this work. This result enforces the added value of an egocentric dataset, as in the top-view it was observed that in many frames, important information for the gesture recognition module was either occluded or out of the frame.

The results presented are compared to the work of Coupeté et al. (2016) that used a Hidden Markov Models gesture recognition engine, in a HRC assembly scenario. The authors in this work, deployed Nearest Neighbors (k-NNs), geodesic distances, as well as Hidden Markov Models, to perform gesture recognition, reaching recognition accuracy results of 85%, with a split of the training and testing data using the 80:20 method, while when testing with unknown operators, the accuracy results concluded to an accuracy of 80% in total. The method presented in this specific paper, outperforms the recognition results of Coupeté et al. (2016), showing very satisfying results.

## 6. Control of the Robot and Evaluation of HRC Scenario

The cobot used in this scenario was the UR3^8^ robotic arm from Universal Robots. The external parts that were used for grasping the cards and for the introduction of physical interaction were from ROBOTIQ (gripper: 2F-140^9^ & force torque sensor: FT-300-S^10^). For the control of the robotic arm, Robot Operating System (ROS) was used. Official ROS packages were used, in this instance, both for the control of the robotic arm (UR3^11^) and for the control of external parts (gripper & force sensor^12^).

As mentioned previously, during the execution of every experiment, there were two different types of robot goal points. First of all, there were the predefined points, like the waiting position or the handover position in the experiments “*Physical Interaction*” and “*Gesture Recognition*”. On the other hand, when pose estimation was inserted, goal points that were estimated on-the-fly were sent to the robot. ROS provides plenty of libraries for the control of the robot. One of them, named ActionLib was used to allow the motion of the robot through a series of predefined poses. To be more specific, it takes a series of robot poses to form a ROS action. To achieve tasks using actions, the notion of a goal that can be sent to an ActionServer by an ActionClient is introduced. The goal is a PoseStamed message that contains information about where the robot should move within its environment. For each position, it computes the inverse kinematics solution to find the joint angles corresponding to the end effector position. Through this procedure, it creates a smooth trajectory and passes it to the drivers of the robot for execution. For experiments with pose estimation MoveIt^13^, a motion planning framework named Open Motion Planning Library (OMPL) was used. From the aforementioned pose estimation procedure, the position of the operator's hand was perceivable and was sent to the robot in the Cartesian space. Therefore, for the specific motion of the robot, the Cartesian path was computed using the MoveIt framework, with specific constraints (same orientation of end effector and safety restriction of velocity). Cartesian planning supports a type of constraint that keeps the robot end effector upright, in order to reduce the possibility of injuring the operator. Cartesian path planning, through the MoveIt framework, satisfied the use-case constraints, as the end effector moved along a straight line, using waypoints interpolation.

Common metrics derived from the literature were used for the evaluation of the proposed Human-Robot Interaction (HRI) system as a whole, the effectiveness of the cobot, and the opinion of the human operators about the Human-Robot (HR) interaction. The evaluation of the system as a whole was able to be measured by specific metrics, such as the efficiency of the robotic arm. This included the time it took for the cobot to move, in relation to the time that the whole routine needed. Had this been extremely small, then this would have revealed that the specific cobot could be used in two assembly lines in parallel, thus speeding up the production process. The evaluation of the effectiveness of the robot was able to be measured by metrics such as neglect tolerance (NT), which is concerned with the amount of time that a human can ignore a cobot, and also robot attention demand (RAD), which measures the attention that the cobot demands from the operator, depending on the degree of Interaction Effort (IE) that is expected from the user. The smaller this number is, the more realistic the interaction between the human and the cobot is.

The NASA Task Load Index (TLX)^14^, is widely used as a subjective workload assessment tool, which rates perceived workload (both mental and physical) in order to assess a task. A version adapted to the specific use case was implemented, in order to evaluate the workload of the task of screwing of electronic cards on a frame. In addition, for every experiment, users were questioned about the relationship that was developed between the robotic arm and them. Finally, users responded concerning which experiment provided the most natural and realistic collaboration.

## 7. Results

Every experiment was executed twice by 14 operators (the group consists of 4 women and 10 men, aged from 23 to 44 with little and medium experience of the execution of TV assembly). During each execution, the operator followed a particular sequence of actions. Initially, s/he asked for the first green card. The robotic arm brought it and the operator checked to see whether the card was functional or not. S/he informed the robotic arm, concerning the functionality of the card, and it reacted accordingly. When the operator had a functional card, s/he started screwing it in place. When the operator was finished, s/he asked for the second card and the robotic arm brought it. The same sequence of actions was executed until both cards were screwed onto the TV panel.

**Figure 10:**
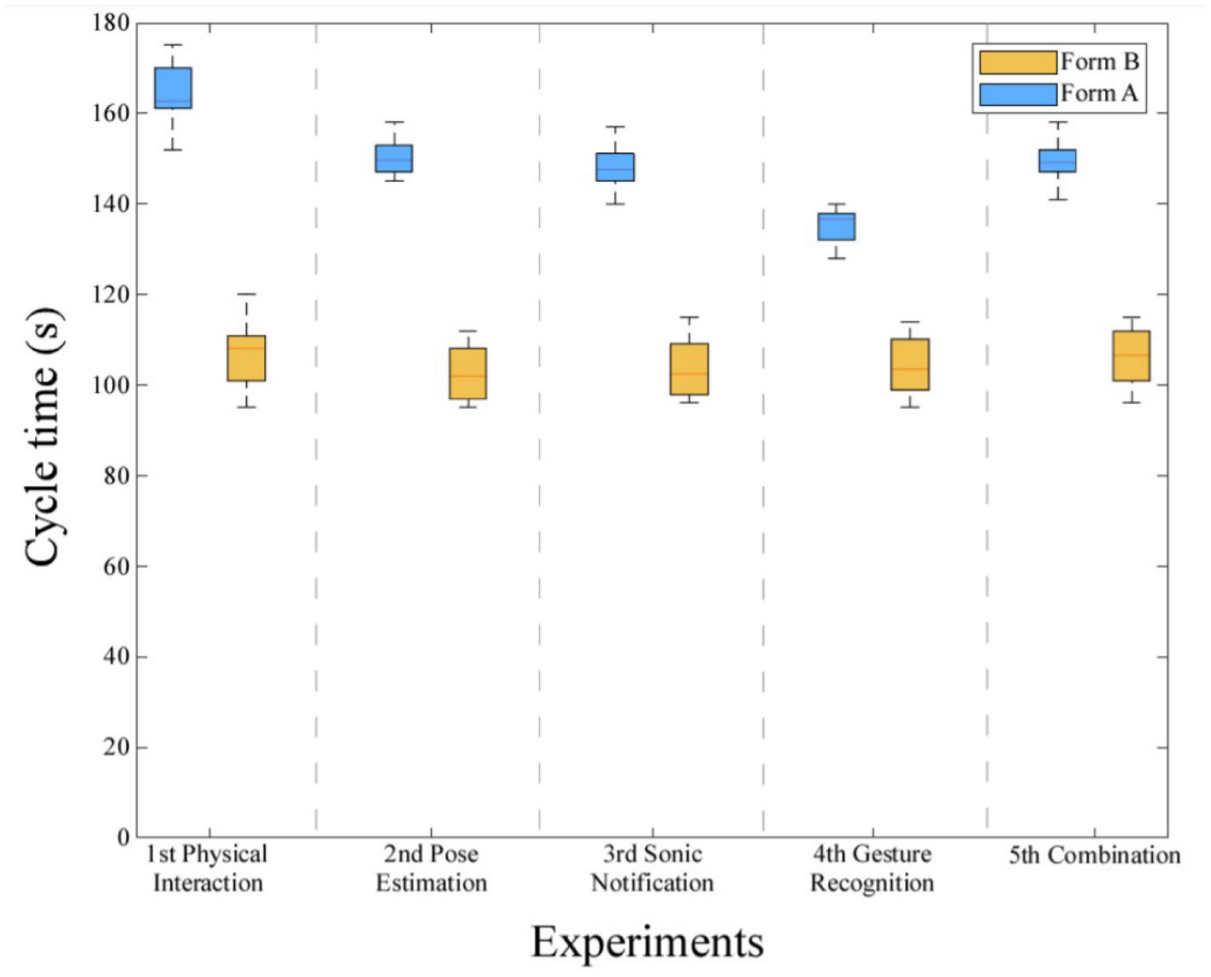
Dynamic cycle time depending on the sequence. Form A: Experiments where the first card of each kind is non-functional. Form B: Experiments where every card is functional.

In the first execution of each experiment, for each operator, the first card of both types (green and gold) was deliberately not functional. As mentioned before, the operator had to inform the robotic arm about the functionality of the electronic cards, depending on the type of interaction that was used in each experiment. In the second execution, every card (of both types) delivered was functional. The purpose of these two types of experiments was to present the adaptation of the robotic arm with a predicted interruption in the procedure. The cycle time for each experiment is presented in Figure 10 by a whisker plot showing and comparing distributions. Experiments with non-functional cards of both kinds are referred to as Form A and the ones with both cards functional are referred to as Form B. A one-way ANOVA for experiments of Form B revealed that there is not a statistically significant difference in cycle time between different types of interaction [*F*(between groups df, within groups df) = [0,29], *p* = [0,88]]. This can be justified as the time of interaction is small and the main parameter that affects the cycle time is the duration of card-screwing operation. However, the questionnaires, which are presented later, proved that the insertion of gesture recognition and pose estimation improves the sense of collaboration and reduce the motion of every user. A second one-way ANOVA test for experiments of Form A is executed and confirms that cycle time changes significantly for different types of interaction (*F* = [10,71], *p* = [9,91e-06]). During the execution of the experiments of Form A, the completion of the routine, when gesture recognition was implemented, lasted 20% less than the experiment “*Physical Interaction*” and about 13% less than the experiments where pose estimation was used.

Figure 10 presents the adaptation of the average cycle time of the routine, depending on the sequence followed. The cycle time of the routine is dynamic and from Figure 10 one more interesting result appears. The fastest execution of the routine takes place when gesture recognition is used as the means of interaction between the operator and the robotic arm. Furthermore, it is important to mention that in Figure 10, the gap between the cycle time of the experiments of Form A and B appears to be about 20% less in the experiment “*Gesture Recognition*”. This metric is an indication that the implementation of gesture recognition in this HRC scenario can reduce the cycle time of the routine, even though predicted or unpredicted incidents occur.

**Figure 11:**
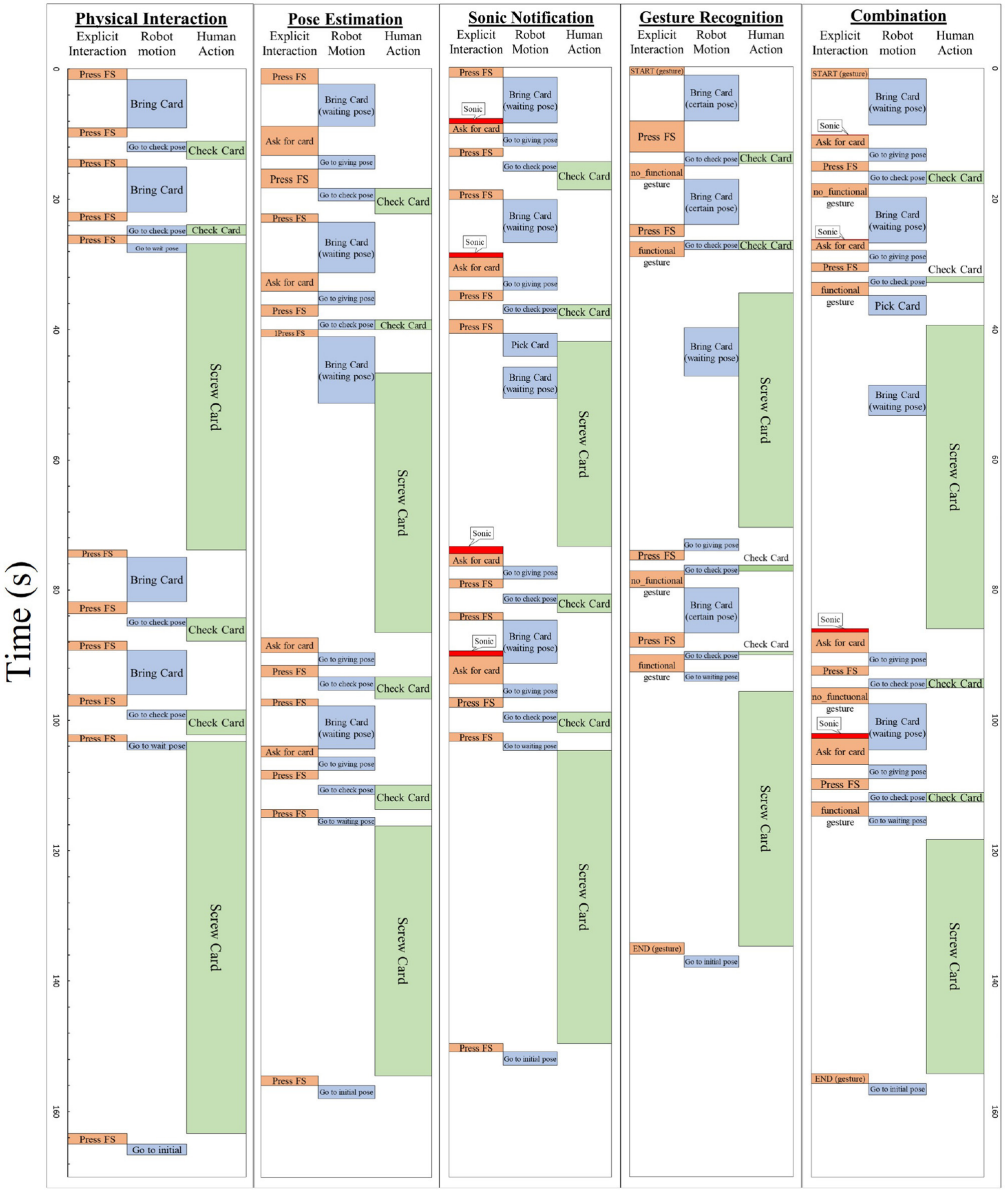
Average timeline of each experiment (Form A).

Figure 11 presents the average timeline of execution of each experiment of Form A. The purpose of this figure is the presentation of the task of each participant in this collaboration and the way that they interact. By way of comparison of each of the interaction types used, the response time for each interaction is given. Response time, in order to facilitate this comparison, is defined as the time from the beginning of the motion of the operator up to the moment that the robot starts moving. The average response time of Physical Interaction (PI) in all experiments is 1.63 s, as compared with 2.87 s, which is the response time for Pose Estimation. Gesture recognition is located between these two and amounts to 2.01 s. Despite the fact that PI seems to display the best response time, in Figure 11 the average cycle time of the experiment “*Physical Interaction*” seems to be greater. This can be explained by the fact that the robot interacts more implicitly and less explicitly (i.e., through direct commands from the operator).

In the experiment “*Physical Interaction*”, the operator has to stop his routine and inform the robot about his current action (by pressing the button) which is replaced by recognition of professional gestures and pose estimation in later experiments. These two modules make the execution of the experiment faster. In the case of the experiments “*Pose Estimation*” and “*Sonic Notification*”, the information about the completion of screwing each card is provided to the robotic arm by the position of the operator's hand (the operator lets go of the screwdriver) and in the case of gesture recognition, the robotic arm is constantly provided with information about the actions that are being executed by the operator. In every experiment, there is a particular sequence of actions that is followed by the operator, which is a safeguard for the smooth execution of the routine.

In Table 3, the metrics that are used for the evaluation of the HRC scenario are presented. Initially, the efficiency of the robot (i.e., the percentage of time that the robot moves while running a program-routine) is measured. During the execution of experiment 1 and 4, the handover position is predefined and the time that the robot moves during the execution of the experiment doesn't change (it is represented by an ^*^ symbol in Table 3). The control box of the robotic arm certifies that while the robotic arm is in motion, the power demanded is approximately 100 W, whereas during the time that the robotic arm remains motionless, it is about 75 W. Thus, this metric informs the operator concerning the time during which the robot is moving and, therefore, concerning the power demands of the robot. As the cycle time of the routine of the experiment “*Gesture Recognition*” is lower and the efficiency of the robot remains at the same level, the motion time of the robot is less, compared to the other experiments. However, by adding the gesture recognition module to within the scenario, and thus a new computer that exploits its GPU to almost its maximum capacity, as well as a camera that provides streaming in real-time, this increases the total power demand by 60 W. As the routine for the whole TV assembly scenario using gesture recognition lasts 136 s, this means an increase of 2.28 Wh for every TV assembled.

**Table 3:** Average of HRC metrics for each experiment. Neglect Tolerance (s), Interaction effort (s), Robot Attention Demand (RAD), and Efficiency of the robot(%) (^*^no spatial adaptation).

	**Neglect tolerance**	**Interaction effort**	**RAD**	**Efficiency of the robot**
Physical interaction	118.9 (σ: 5.4)	15.5 (σ: 3.2)	0.12 (σ: 0.001)	23^*^
Pose estimation	90.6 (σ: 3.8)	27.6 (σ: 4.1)	0.23 (σ: 0.001)	31 (σ: 0.3)
Sound notification	88.8 (σ: 4.1)	30.9 (σ: 4.7)	0.26 (σ: 0.002)	30 (σ: 0.8)
Gesture recognition	61.4 (σ: 3.2)	23.1 (σ: 2.6)	0.27 (σ: 0.001)	28^*^
Combination	88.1 (σ: 3.6)	30.0 (σ: 4.0)	0.25 (σ: 0.001)	29 (σ: 0.4)

Neglect Tolerance (NT) and Interaction Effort (IE), that were mentioned previously, are also presented in Table 3 with their standard deviation in parenthesis. Robot Attention Demand (RAD) is calculated using the following equation:


(7) $$\begin{array}{l} RAD=\frac{IE}{NT+IE} \end{array}$$

This metric provides us with information about how many times the operator has paid attention to the robot and has provided it with commands concerning the next step of the routine. As NT contains the time when the screwing of cards is executed, RAD is a metric that depends on the rhythm of each operator. The greater the value of NT is, the less rich information the robot receives about the human's actions and intentions. Moreover, the larger the RAD, the more the robot is able to understand and adapt to its partner. The average, as presented in Table 3 and its standard deviation in parenthesis, shows that RAD is stable among the last four experiments, despite the fact that NT is significantly smaller during the experiments in which gesture recognition is implemented. The reason that RAD is smaller during the execution of the experiment “*Physical Interaction*” is that the NT is greater, as the operator interacts only explicitly with the robotic arm.

During the execution of the experiments without spatial adaptation (*SA*), the operator receives the cards from a particular handover position (*PHP*). The KPI that is proposed in equation 8 indicates the percentage of robot spatial adaptation in the case of every operator. For the calculation of the KPI, the distance from the waiting point (*WP*), to the particular handover position for the experiments that is stable, is compared to the adaptable handover position (*AHP*) for the experiments in which pose estimation is implemented. Distances are measured in centimeters. The higher the adaptation rate, the greater the effort that was demanded of the operator during the experiments, without spatial adjustment. As Table 4 shows, operators 3 and 12 asked for the card to be brought closer to the particular handover position and as a result the robotic arm had to adapt less than for the other operators. This KPI could also be useful for discovering the position that each individual user prefers as the handover position.


(8) $$\begin{array}{l} SA\left(\%\right)=\frac{\left\|AHP-WP\|-\|PHP-WP\right\|}{\left\|PHP-WP\right\|} \end{array}$$

Where *SA*: spatial adaptation, *AHP*: adapted handover position, *WP*: waiting point and *PHP*: particular handover position.

**Table 4:** Spatial adaptation (%) of each operator.

**Operators**	**1**	**2**	**3**	**4**	**5**	**6**	**7**	**8**	**9**	**10**	**11**	**12**	**13**	**14**
Spatial adaptation	40.2	33.9	19.6	45.6	36.5	40.2	41.9	39.5	27.5	28.9	33.1	21.5	39.2	23.9

Human factors (or ergonomics) are defined by ISO 26800 as the “scientific discipline concerned with the understanding of interactions among human and other elements of a system, and the profession that applies theory, principles, data, and methods to design in order to optimize human well-being and overall system performance”. In order to achieve an optimal level of collaboration, it is essential to take into account the opinion of the human involved in operations with the robot. To evaluate the execution of the experiments users responded to two different questionnaires. Initially the workload of the TV assembling task was estimated through the NASA-TLX. This tool consists of a questionnaire with six items for evaluation: mental demand, physical demand, temporal demand, effort, frustration and performance.

11 out of 14 participants replied that the task they undertook was neither physically nor mentally demanding. In addition, none of the them felt that the pace of the task was hurried. Thus, the reason that a cobot is used to substitute a human operator for this task is the need for repeatability and the fact that a cobot can not only repeat the same task many times, but can perform the task precisely and fast. Every participant was able to accomplish all the experiments and responded that they did not find it difficult to interact with the robot and understand its reactions. Due to the inexperience of some users, some errors occurred during the execution of the experiments; however, this did not affect the accomplishment of the task, as the robotic arm was following a particular sequence of actions.

**Figure 12:**
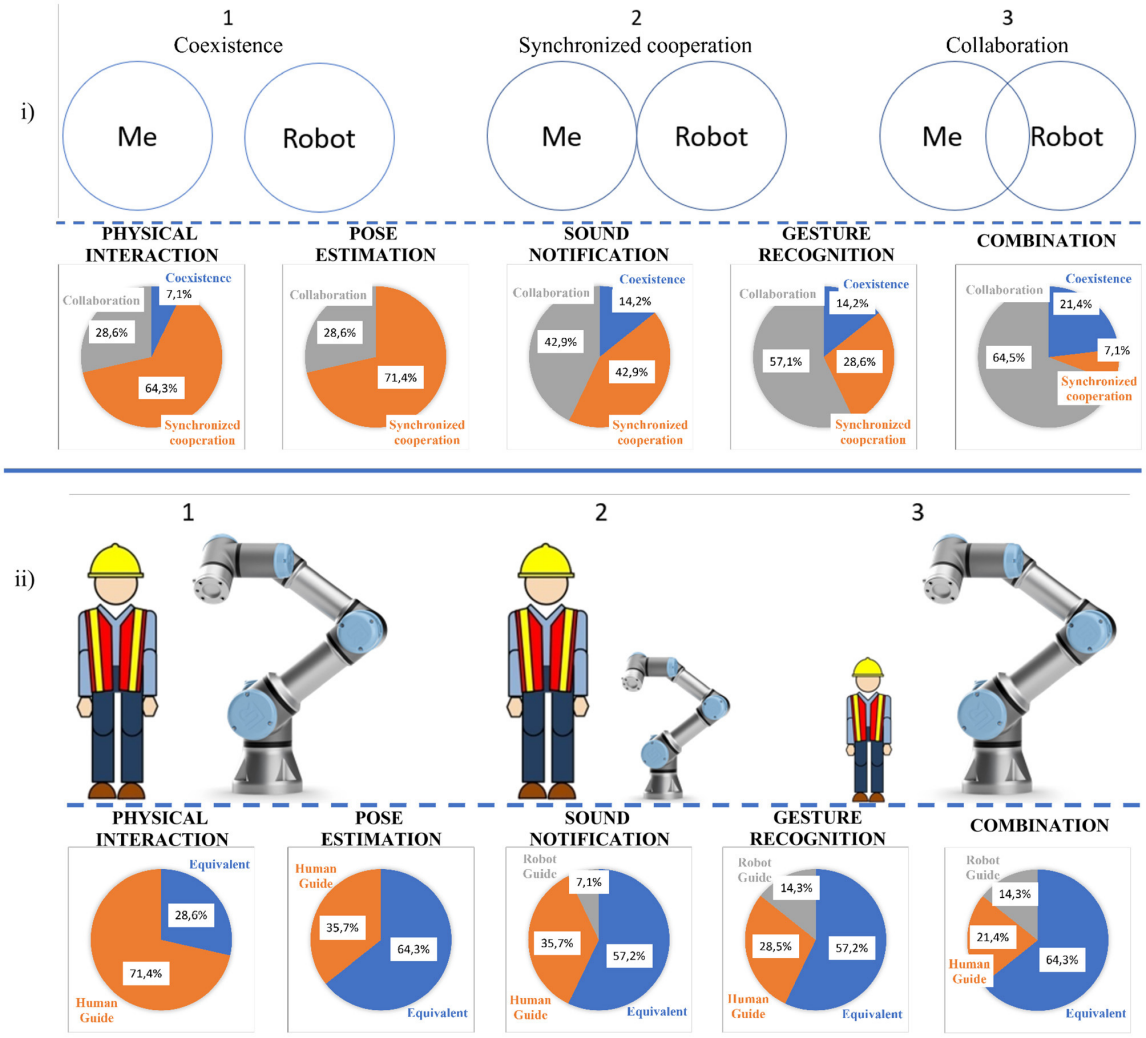
**(i)** Categories of Human-Robot Interaction, **(ii)** Relationship between robot and human operator.

In addition, the participants were asked to categorize the type of HRI of each experiment and to characterize the relationship between the robot and the operator during the execution of each experiment. In section 2.1, the categories of HRI are analyzed (Coexistance, Synchronized Cooperation and Collaboration). In the first part of Figure 12, the types, from among which the participants chose the category of HRI, are presented. The majority of the respondents thought that the implementation of gesture recognition in the experiment “*Gesture Recognition*” and “*Combination*” strengthened the sense of collaboration, while they felt that the first three experiments belonged to the category of synchronized cooperation. All the participants considered that with only “*Physical Interaction*”, the robotic arm was simply following the human operator, which led to slower execution of the task. Finally, as was mentioned before, the aim of this research is to convert the robot from a useful machine to a real collaborator. When only physical interaction was used (1st experiment) most of the users felt that the robot had a supporting role. However, 9 out of 14 participants declared that the insertion of “*Pose estimation*” or “*Gesture Recognition*” made them feel that their contribution to the task was equal to that of the robot.

## 8. Discussion

The proposed methodology and the experiments concerning the contribution of different modalities to an HRC scenario, concludes by showing great potential for the future. Both hypotheses that were defined at the beginning of this work, are evaluated. Through the experiments performed, it was validated that on what concerns the temporal adaptation of the robotic arm, the insertion of gesture recognition reduces the cycle time of the routine of every operator (by 20% on average), adding a relatively small increase in energy consumption by the system. The second hypothesis was concerned with the implementation of pose estimation in order to achieve the spatial adaptation of the robotic arm. According to the results collected, the hypotheses presented are both valid. For 9 of the 14 operators, the percentage of spatial adaptation is more than 30%, which shows the importance of this modality regarding reducing the operator's effort.

Concerning the different modalities, gesture recognition is proved to be capable of accelerating an assembly line and of providing the human operator with a sense of true cooperation with the cobot and not just coexistence. Meanwhile, pose estimation offers the prospect of converting the cobot to a partner who adapts to every operator. A significant observation for pose estimation is that robot attention demand is increasing while the average motion time of the robot decreases in contrast to physical interaction. The argument given above proves that the cobot possesses more information about the human operator and as a result it moves less during the routine, as it can predict human's motions.

In both gesture recognition and pose estimation modules, the response time is satisfactory, within a challenging task, that facilitates the spatiotemporal adaptation. A great improvement in the accuracy of gesture recognition was noted after the implementation of transfer learning, proving that the initial amount of acquired data was not sufficient, even after a few sessions of recording. 3DCNNs have to be robust and extract confident results, even in real-time, with operators that the network has not been trained with. Egocentric gesture recognition might be a challenging task, but it can lead to impressive results, independent of anthropometric characteristics and clothing. The most important observation that the gestural module provided, was the fact that it can be used in a real-life assembly line with great results, without retraining the network each time that a new human operator was introduced to it. Even though handling data from an egocentric point of view was a challenging task in order for an accurate classification to be performed, and for the safety of the human operator to be ensured, it provided great potential for the future. Apart from this, the TV assembly dataset created can be enriched with more classes in an egocentric view from different professional environments, in order for the proposed approach to respond to different professional setups.

The operator's sense of collaboration with the cobot improved significantly because of the sonic notification. It could be enriched with many different kinds of messages; however, due to the fact that this use-case is intended for an industrial environment, simplicity has to be preserved. Furthermore, the existence of many different sonic notifications could create comprehension problems in the case of many parallel assembly lines. The questionnaire validated the fact that the task was neither mentally nor physically demanding. The reason that it was used for this research was its repetitiveness, because robots tend to take over the dull, dirty, dangerous and dear (i.e., costly) tasks from humans, otherwise known as the 4 Ds of robotization. Finally, according to the answers of the participants, the implementation of pose estimation made them feel that they participated equally with the robot in the routine of TV assembly, while gesture recognition enhanced the sense of collaboration in contrast to synchronized cooperation.

## 9. Conclusion and Future work

In this paper, an HRC scenario is defined and different modalities are evaluated concerning the cycle time of the execution of a TV assembly routine and the naturalness of this collaboration, according to the human operators. The insertion of gesture recognition accelerates the execution of the proposed routine by about 20%, reducing, in parallel, the effort required of the operator, in order to perform.

In this research, a new KPI regarding spatial adaptation is proposed and shows that the insertion of a cobot with a dynamic spatial profile that adjusted to the operators, changes the handover position of the experiment by up to 40%. The ergonomic parameters of a task can be analyzed and the robot adjusts its motion not only to avoid collisions with the operator, but also in order to ergonomically improve the pose of the operator during the execution of their task.

Moreover, this paper opens up potential for investigating industrial HRC scenarios and proposing intelligent and efficient solutions on the road to Industry 4.0. This research could have been enriched with experiments executed by professional users from the industry; however, due to the conditions imposed by Covid-19 restrictions, this was impossible. Our future work will be focused on the upgrading of the robot's perception of the user and their environment, with an aim to improving their collaboration. To this end, the way that the robot can make best use of pose estimation is investigated. Finally, the fact that the robot is able to perceive through pose estimation, and to follow the position and every action of the operator in real time, undoubtedly improves their collaboration and further facilitates the insertion of robots in common industrial work-spaces with human operators.

## Data Availability Statement

The raw data supporting the conclusions of this article can be made available by the authors upon request.

## Ethics Statement

Ethical review and approval was not required for the study on human participants in accordance with the local legislation and institutional requirements. The patients/participants provided their written informed consent to participate in this study.

## Author Contributions

DP: leader of cobot control, the pose estimation module, and its integration with the cobot, and co-leader of the experimental protocol, architecture, and realization. GS: leader of the recordings of the used dataset, segmentation, and labeling, defined the gestural vocabulary, and interpreted the recognition results, developer of the gesture recognition module and its integration with the cobot, and co-leader of the experimental protocol, architecture, and realization. SM: conceptualization of the methodology and definition of the scientific and industrial needs to be addressed with respect to human-robot collaboration, machine learning, and pose estimation. All authors contributed to the article and approved the submitted version.

## Funding

The research leading to these results has received funding from the EU Horizon 2020 Research and Innovation Programme under Grant Agreement No. 820767, CoLLaboratE project.

## Conflict of Interest

The authors declare that the research was conducted in the absence of any commercial or financial relationships that could be construed as a potential conflict of interest.

## Publisher's Note

All claims expressed in this article are solely those of the authors and do not necessarily represent those of their affiliated organizations, or those of the publisher, the editors and the reviewers. Any product that may be evaluated in this article, or claim that may be made by its manufacturer, is not guaranteed or endorsed by the publisher.

## Footnotes

^1^ https://ifr.org/

^2^ https://www.intel.com/content/dam/www/public/us/en/documents/pdf/the-need-for-enabling-touchless-technologies-whitepaper.pdf

^3^ http://roboticsandautomationnews.com/2017/03/04/bmw-shows-off-its-smart-factory-technologies-at-its-plants-worldwide/11696/

^4^ https://en.wikipedia.org/wiki/Kinect

^5^ https://developer.leapmotion.com/

^6^ https://github.com/CMU-Perceptual-Computing-Lab/openpose

^7^ https://gopro.com/en/fr/shop/cameras/hero9-black/CHDHX-901-master.html?gclsrc=aw.ds&&gclid=Cj0KCQiAjKqABhDLARIsABbJrGnysxveH64ikG8aUbTJACoVucx259TEujqz_3cDlwFAZuE5Yhgi5zKoaAmbHEALw_wcB

^8^ https://www.universal-robots.com/products/ur3-robot/

^9^ https://robotiq.com/products/ft-300-force-torque-sensor

^10^ https://robotiq.com/products/2f85-140-adaptive-robot-gripper

^11^ https://github.com/UniversalRobots/Universal_Robots_ROS_Driver

^12^ https://github.com/ros-industrial/robotiq

^13^ https://moveit.ros.org/

^14^ https://humansystems.arc.nasa.gov/groups/tlx/
